# Flavokavain B from the rhizome of *Alpinia mutica* Roxb

**DOI:** 10.1107/S1600536810041395

**Published:** 2010-10-20

**Authors:** Hasnah Mohd Sirat, Nor Akmalazura Jani, Hazrina Hazni, Khalijah Awang, Seik Weng Ng

**Affiliations:** a Department of Chemistry, Universiti Teknologi Malaysia, 81310 Skudai, Malaysia; b Department of Chemistry, University of Malaya, 50603 Kuala Lumpur, Malaysia

## Abstract

The title compound [systematic name: (*E*)-1-(2-hydroxy-4,6-dimethoxyphenyl)-3-phenylprop-2-en-1-one],  C_17_H_16_O_4_, has an aromatic ring at both ends of the –CH= CH–C(=O)– fragment with the –CH=CH– bond in a *trans* configuration. The phenyl ring is nearly coplanar with this fragment [dihedral angle 4.8 (3) °] as is the hy­droxy­ldimeth­oxy­lphenyl unit [dihedral angle 6.3 (3) °]. The hy­droxy group is the donor in an intra­molecular hydrogen bond to the double-bonded O atom.

## Related literature

For the isolation and spectroscopic characterization of the title compound, see: Flores *et al.* (2007 ▶); Xuan *et al.* (2008 ▶).
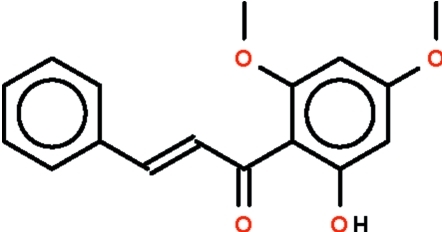


## Experimental

### 

#### Crystal data

C_17_H_16_O_4_
*M* *_r_* = 284.30
Orthorhombic, 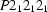

*a* = 4.9668 (10) Å
*b* = 12.305 (3) Å
*c* = 22.552 (5) Å
*V* = 1378.3 (5) Å^3^
*Z* = 4
Mo *K*α radiation
μ = 0.10 mm^−1^
*T* = 100 K
0.40 × 0.10 × 0.05 mm

#### Data collection

Bruker SMART APEX diffractometer
10723 measured reflections
1449 independent reflections
1162 reflections with *I* > 2σ(*I*)
*R*_int_ = 0.089

#### Refinement

*R*[*F* ^2^ > 2σ(*F* ^2^)] = 0.049
*wR*(*F* ^2^) = 0.128
*S* = 1.09
1449 reflections
197 parameters
1 restraint
H atoms treated by a mixture of independent and constrained refinement
Δρ_max_ = 0.20 e Å^−3^
Δρ_min_ = −0.27 e Å^−3^

### 

Data collection: *APEX2* (Bruker, 2009 ▶); cell refinement: *SAINT* (Bruker, 2009 ▶); data reduction: *SAINT*; program(s) used to solve structure: *SHELXS97* (Sheldrick, 2008 ▶); program(s) used to refine structure: *SHELXL97* (Sheldrick, 2008 ▶); molecular graphics: *X-SEED* (Barbour, 2001 ▶); software used to prepare material for publication: *publCIF* (Westrip, 2010 ▶).

## Supplementary Material

Crystal structure: contains datablocks global, I. DOI: 10.1107/S1600536810041395/fl2321sup1.cif

Structure factors: contains datablocks I. DOI: 10.1107/S1600536810041395/fl2321Isup2.hkl

Additional supplementary materials:  crystallographic information; 3D view; checkCIF report

## Figures and Tables

**Table 1:** Hydrogen-bond geometry (Å, °)

*D*—H⋯*A*	*D*—H	H⋯*A*	*D*⋯*A*	*D*—H⋯*A*
O2—H2⋯O1	0.85 (1)	1.65 (2)	2.455 (4)	157 (4)

**Fig. 1.**
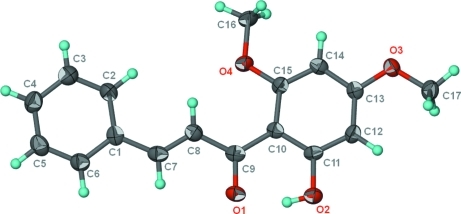
Thermal ellipsoid plot (Barbour, 2001) of the C17H16O4 at the 70% probability level; hydrogen atoms are drawn as spheres of arbitrary radius.

**Fig. 2.**
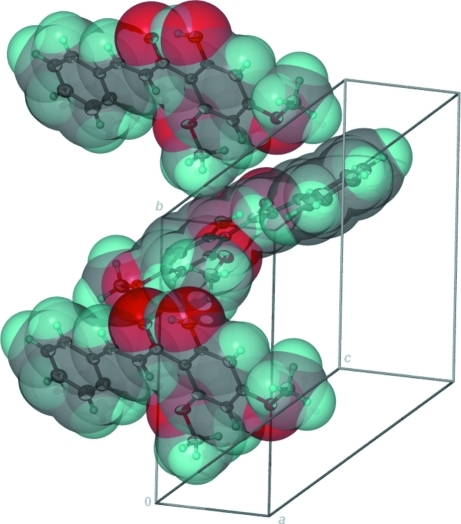
Close approach of ajacent molecules.

## supplementary crystallographic information

### Comment

*Alpinia mutica* is a perennial herb (Zingiberaceae) endemic to southern parts of Malaysia. It is also cultivated as an ornamental plant and the rhizomes are used as an herb for strengthening the stomach. Among the compounds isolated this herb is Flavokavain B, whose structure was elucidated by spectrospic methods (Flores *et al.*, 2007; Xuan *et al.*, 2008) and whose x-ray structure is reported here. The chalcone (Scheme I) has aromatic rings at either ends of the –CH═ CH–C(═O)– linkage; the -CH=CH- double bond has a *trans* configuration. The phenyl ring is nearly coplanar with this fragment [dihedral angle 4.8 (3) °] as is the hydroxyldimethoxylphenyl ring [dihderal angle 6.3 (3) ° (the dihedral angle between the two rings is 10.0 (2)°) (Fig. 1). The hydroxy group is donor in an intra-molecular H-bond bond with the double-bond oxygen atom of the fragment. Other than a close contact of 3.04 Å between O2 and C16, there are no important intermolecular contacts (Fig. 2).

The compound has been previously isolated and characterized by NMR spectroscopy (Flores *et al.*, 2007; Xuan *et al.*, 2008).

### Experimental

The rhizome of *Alpinia mutica* was collected from Pontian, Johor, Malaysia. A voucher specimen was deposited at the Herbarium of the Departmentof Botany, Universiti Putra Malaysia. The *n*-hexane crude extract of the rhizome (8.97 g) was subjected to silica-gel chromatography and was eluted out by using a gradient mixture of petroleum ether, ethanol and methanol. Twenty three fractions were collected, which were then separated by TLC to afford eight fractions. The sixth fraction was subjected to silica-gel column chromatography to give the title compound (petroleum ether: ether 4/1)which  was recrystallized from petroleum ether/ether to afford faint yellow-orange crystals suitable for data collection.

### Refinement

Carbon-bound H-atoms were placed in calculated positions (C—H 0.95–0.98 Å) and were included in the refinement in the riding model approximation, with *U*(H) set to 1.2–15*U*(C).

The hydroxy H-atom was located in a difference Fourier map, and was refined with the O–H distance restrained to 0.84±0.01 Å; its temperature factor was refined.

In the absence of heavy atom, Some 976 Friedel pairs were merged.

### Crystal data

**Table:**

C_17_H_16_O_4_	*F*(000) = 600
*M**_r_* = 284.30	*D*_x_ = 1.370 Mg m^−^^3^
Orthorhombic, *P*2_1_2_1_2_1_	Mo *K*α radiation, λ = 0.71073 Å
Hall symbol: P 2ac 2ab	Cell parameters from 1135 reflections
*a* = 4.9668 (10) Å	θ = 3.2–21.7°
*b* = 12.305 (3) Å	µ = 0.10 mm^−^^1^
*c* = 22.552 (5) Å	*T* = 100 K
*V* = 1378.3 (5) Å^3^	Prism, faint yellow
*Z* = 4	0.40 × 0.10 × 0.05 mm

### Data collection

**Table:**

Bruker SMART APEX diffractometer	1162 reflections with *I* > 2σ(*I*)
Radiation source: fine-focus sealed tube	*R*_int_ = 0.089
graphite	θ_max_ = 25.0°, θ_min_ = 1.8°
ω scans	*h* = −5→5
10723 measured reflections	*k* = −14→14
1449 independent reflections	*l* = −26→25

### Refinement

**Table:**

Refinement on *F*^2^	Secondary atom site location: difference Fourier map
Least-squares matrix: full	Hydrogen site location: inferred from neighbouring sites
*R*[*F*^2^ > 2σ(*F*^2^)] = 0.049	H atoms treated by a mixture of independent and constrained refinement
*wR*(*F*^2^) = 0.128	*w* = 1/[σ^2^(*F*_o_^2^) + (0.0736*P*)^2^ + 0.021*P*] where *P* = (*F*_o_^2^ + 2*F*_c_^2^)/3
*S* = 1.09	(Δ/σ)_max_ = 0.001
1449 reflections	Δρ_max_ = 0.20 e Å^−^^3^
197 parameters	Δρ_min_ = −0.26 e Å^−^^3^
1 restraint	Extinction correction: *SHELXL*, Fc^*^=kFc[1+0.001xFc^2^λ^3^/sin(2θ)]^-1/4^
Primary atom site location: structure-invariant direct methods	Extinction coefficient: 0.026 (5)

### Fractional atomic coordinates and isotropic or equivalent isotropic displacement parameters (Å^2^)

**Table:**

	*x*	*y*	*z*	*U*_iso_*/*U*_eq_	
O1	0.5688 (6)	0.96830 (19)	0.22125 (11)	0.0250 (7)	
O2	0.3291 (6)	0.9379 (2)	0.12751 (11)	0.0260 (7)	
H2	0.440 (7)	0.958 (4)	0.1541 (14)	0.048 (15)*	
O3	−0.3912 (5)	0.68445 (19)	0.12028 (10)	0.0245 (7)	
O4	0.0995 (6)	0.72577 (18)	0.29718 (11)	0.0272 (7)	
C1	0.7327 (8)	0.9257 (3)	0.40417 (17)	0.0228 (9)	
C2	0.5796 (9)	0.8616 (3)	0.44201 (16)	0.0283 (10)	
H2A	0.4369	0.8191	0.4262	0.034*	
C3	0.6311 (10)	0.8586 (3)	0.50210 (18)	0.0349 (10)	
H3	0.5230	0.8150	0.5274	0.042*	
C4	0.8406 (9)	0.9191 (3)	0.52537 (17)	0.0323 (11)	
H4	0.8762	0.9177	0.5668	0.039*	
C5	0.9976 (9)	0.9815 (3)	0.48823 (18)	0.0310 (10)	
H5	1.1418	1.0229	0.5042	0.037*	
C6	0.9476 (9)	0.9846 (3)	0.42798 (17)	0.0279 (10)	
H6	1.0594	1.0267	0.4027	0.034*	
C7	0.6751 (8)	0.9328 (3)	0.34039 (17)	0.0257 (9)	
H7	0.7994	0.9723	0.3167	0.031*	
C8	0.4658 (8)	0.8891 (3)	0.31275 (16)	0.0256 (9)	
H8	0.3381	0.8493	0.3354	0.031*	
C9	0.4237 (8)	0.8998 (3)	0.24859 (16)	0.0230 (9)	
C10	0.2169 (8)	0.8393 (3)	0.21647 (16)	0.0196 (8)	
C11	0.1737 (8)	0.8639 (3)	0.15591 (16)	0.0208 (9)	
C12	−0.0285 (8)	0.8174 (3)	0.12220 (16)	0.0230 (9)	
H12	−0.0586	0.8392	0.0823	0.028*	
C13	−0.1857 (8)	0.7377 (3)	0.14850 (16)	0.0213 (9)	
C14	−0.1454 (8)	0.7058 (3)	0.20702 (16)	0.0221 (8)	
H14	−0.2544	0.6504	0.2238	0.027*	
C15	0.0513 (8)	0.7543 (3)	0.24038 (15)	0.0211 (9)	
C16	−0.0619 (9)	0.6404 (3)	0.32205 (16)	0.0298 (10)	
H16A	−0.0115	0.6292	0.3636	0.045*	
H16B	−0.0320	0.5731	0.2998	0.045*	
H16C	−0.2525	0.6606	0.3197	0.045*	
C17	−0.4398 (9)	0.7113 (3)	0.05989 (15)	0.0287 (10)	
H17A	−0.5907	0.6679	0.0449	0.043*	
H17B	−0.2783	0.6957	0.0364	0.043*	
H17C	−0.4839	0.7887	0.0567	0.043*	

### Atomic displacement parameters (Å^2^)

**Table:**

	*U*^11^	*U*^22^	*U*^33^	*U*^12^	*U*^13^	*U*^23^
O1	0.0269 (15)	0.0177 (12)	0.0304 (15)	−0.0032 (12)	0.0027 (13)	0.0014 (11)
O2	0.0313 (17)	0.0199 (13)	0.0266 (16)	−0.0038 (13)	0.0000 (14)	0.0034 (12)
O3	0.0254 (15)	0.0218 (13)	0.0263 (14)	−0.0010 (12)	−0.0042 (13)	−0.0008 (11)
O4	0.0393 (17)	0.0180 (12)	0.0243 (14)	−0.0075 (13)	−0.0017 (13)	0.0058 (11)
C1	0.026 (2)	0.0154 (19)	0.027 (2)	0.0059 (17)	−0.0030 (18)	−0.0031 (16)
C2	0.031 (2)	0.0254 (19)	0.028 (2)	−0.006 (2)	−0.002 (2)	−0.0019 (17)
C3	0.038 (3)	0.037 (2)	0.030 (2)	−0.006 (2)	0.001 (2)	0.0001 (19)
C4	0.044 (3)	0.026 (2)	0.027 (2)	0.001 (2)	−0.006 (2)	0.0006 (17)
C5	0.035 (3)	0.021 (2)	0.037 (2)	−0.0020 (19)	−0.006 (2)	−0.0040 (17)
C6	0.032 (2)	0.0211 (19)	0.031 (2)	−0.0026 (19)	−0.0017 (19)	0.0021 (17)
C7	0.028 (2)	0.0173 (18)	0.032 (2)	−0.0015 (18)	0.005 (2)	0.0007 (17)
C8	0.030 (2)	0.0205 (19)	0.026 (2)	−0.0034 (17)	−0.0005 (19)	−0.0006 (16)
C9	0.024 (2)	0.0154 (16)	0.029 (2)	0.0021 (17)	0.0054 (19)	−0.0024 (16)
C10	0.022 (2)	0.0140 (17)	0.023 (2)	0.0009 (15)	0.0026 (17)	−0.0038 (15)
C11	0.025 (2)	0.0115 (17)	0.025 (2)	0.0016 (16)	0.0052 (18)	0.0015 (15)
C12	0.030 (2)	0.0202 (19)	0.0191 (19)	0.0022 (17)	−0.0020 (18)	−0.0002 (15)
C13	0.024 (2)	0.0159 (17)	0.025 (2)	0.0029 (16)	−0.0010 (17)	−0.0036 (15)
C14	0.022 (2)	0.0158 (16)	0.029 (2)	0.0009 (16)	0.0002 (18)	0.0005 (15)
C15	0.030 (2)	0.0139 (16)	0.0197 (18)	0.0015 (16)	0.0000 (18)	−0.0011 (14)
C16	0.043 (3)	0.0207 (18)	0.026 (2)	−0.0075 (19)	−0.002 (2)	0.0089 (16)
C17	0.036 (3)	0.027 (2)	0.024 (2)	−0.001 (2)	−0.001 (2)	−0.0043 (16)

### Geometric parameters (Å, °)

**Table:**

O1—C9	1.269 (4)	C7—C8	1.326 (5)
O2—C11	1.354 (4)	C7—H7	0.9500
O2—H2	0.85 (1)	C8—C9	1.468 (5)
O3—C13	1.370 (5)	C8—H8	0.9500
O3—C17	1.422 (4)	C9—C10	1.461 (5)
O4—C15	1.349 (4)	C10—C11	1.415 (5)
O4—C16	1.436 (4)	C10—C15	1.436 (5)
C1—C2	1.389 (5)	C11—C12	1.384 (5)
C1—C6	1.397 (5)	C12—C13	1.386 (5)
C1—C7	1.469 (5)	C12—H12	0.9500
C2—C3	1.380 (5)	C13—C14	1.391 (5)
C2—H2A	0.9500	C14—C15	1.370 (5)
C3—C4	1.383 (6)	C14—H14	0.9500
C3—H3	0.9500	C16—H16A	0.9800
C4—C5	1.378 (6)	C16—H16B	0.9800
C4—H4	0.9500	C16—H16C	0.9800
C5—C6	1.382 (5)	C17—H17A	0.9800
C5—H5	0.9500	C17—H17B	0.9800
C6—H6	0.9500	C17—H17C	0.9800
			
C11—O2—H2	103 (3)	C11—C10—C15	115.5 (3)
C13—O3—C17	117.4 (3)	C11—C10—C9	118.4 (3)
C15—O4—C16	117.5 (3)	C15—C10—C9	126.0 (3)
C2—C1—C6	118.5 (4)	O2—C11—C12	115.6 (3)
C2—C1—C7	121.9 (4)	O2—C11—C10	120.9 (4)
C6—C1—C7	119.6 (4)	C12—C11—C10	123.5 (3)
C3—C2—C1	121.1 (4)	C11—C12—C13	117.8 (3)
C3—C2—H2A	119.4	C11—C12—H12	121.1
C1—C2—H2A	119.4	C13—C12—H12	121.1
C2—C3—C4	119.8 (4)	O3—C13—C12	124.0 (3)
C2—C3—H3	120.1	O3—C13—C14	114.4 (3)
C4—C3—H3	120.1	C12—C13—C14	121.6 (4)
C5—C4—C3	119.7 (4)	C15—C14—C13	120.0 (4)
C5—C4—H4	120.2	C15—C14—H14	120.0
C3—C4—H4	120.2	C13—C14—H14	120.0
C4—C5—C6	120.7 (4)	O4—C15—C14	122.3 (3)
C4—C5—H5	119.6	O4—C15—C10	116.4 (3)
C6—C5—H5	119.6	C14—C15—C10	121.3 (3)
C5—C6—C1	120.1 (4)	O4—C16—H16A	109.5
C5—C6—H6	120.0	O4—C16—H16B	109.5
C1—C6—H6	120.0	H16A—C16—H16B	109.5
C8—C7—C1	126.1 (4)	O4—C16—H16C	109.5
C8—C7—H7	117.0	H16A—C16—H16C	109.5
C1—C7—H7	117.0	H16B—C16—H16C	109.5
C7—C8—C9	122.6 (4)	O3—C17—H17A	109.5
C7—C8—H8	118.7	O3—C17—H17B	109.5
C9—C8—H8	118.7	H17A—C17—H17B	109.5
O1—C9—C10	119.8 (3)	O3—C17—H17C	109.5
O1—C9—C8	117.2 (4)	H17A—C17—H17C	109.5
C10—C9—C8	122.9 (3)	H17B—C17—H17C	109.5
			
C6—C1—C2—C3	2.3 (6)	C15—C10—C11—C12	5.5 (5)
C7—C1—C2—C3	−177.9 (4)	C9—C10—C11—C12	−175.4 (3)
C1—C2—C3—C4	−0.8 (6)	O2—C11—C12—C13	177.2 (3)
C2—C3—C4—C5	−0.4 (6)	C10—C11—C12—C13	−4.0 (5)
C3—C4—C5—C6	0.2 (6)	C17—O3—C13—C12	1.6 (5)
C4—C5—C6—C1	1.3 (6)	C17—O3—C13—C14	−178.6 (3)
C2—C1—C6—C5	−2.5 (6)	C11—C12—C13—O3	−179.6 (3)
C7—C1—C6—C5	177.7 (4)	C11—C12—C13—C14	0.7 (5)
C2—C1—C7—C8	6.2 (6)	O3—C13—C14—C15	−178.9 (3)
C6—C1—C7—C8	−173.9 (4)	C12—C13—C14—C15	0.8 (6)
C1—C7—C8—C9	−179.8 (4)	C16—O4—C15—C14	0.8 (5)
C7—C8—C9—O1	−12.0 (5)	C16—O4—C15—C10	−179.6 (3)
C7—C8—C9—C10	170.8 (4)	C13—C14—C15—O4	−179.6 (3)
O1—C9—C10—C11	−3.8 (5)	C13—C14—C15—C10	0.9 (6)
C8—C9—C10—C11	173.4 (3)	C11—C10—C15—O4	176.6 (3)
O1—C9—C10—C15	175.3 (3)	C9—C10—C15—O4	−2.5 (5)
C8—C9—C10—C15	−7.5 (6)	C11—C10—C15—C14	−3.8 (5)
C15—C10—C11—O2	−175.9 (3)	C9—C10—C15—C14	177.1 (4)
C9—C10—C11—O2	3.3 (5)		

### Hydrogen-bond geometry (Å, °)

**Table:**

*D*—H···*A*	*D*—H	H···*A*	*D*···*A*	*D*—H···*A*
O2—H2···O1	0.85 (1)	1.65 (2)	2.455 (4)	157 (4)
